# A vegan dietary pattern is associated with high prevalence of inadequate protein intake in older adults; a simulation study

**DOI:** 10.1016/j.jnha.2025.100536

**Published:** 2025-03-26

**Authors:** Jos W. Borkent, Pol Grootswagers, Joost Linschooten, Annet J.C. Roodenburg, Marga Ocké, Marian A.E. de van der Schueren

**Affiliations:** a HAN University of Applied Sciences, Department of Nutrition, Dietetics and Lifestyle, Nijmegen, the Netherlands; b Wageningen University, Department of Human Nutrition and Health, Wageningen, the Netherlands; c HAS Green Academy, Department of Food and Industry, 's-Hertogenbosch, the Netherlands; d National Institute for Public Health and the Environment, Bilthoven, the Netherlands

**Keywords:** Protein quality, Protein intake, Simulation study, PDCAAS, Sustainability, Amino acid composition

## Abstract

**Background:**

A more sustainable diet with fewer animal-based products has a lower ecological impact but might lead to a lower protein quantity and quality. The extent to which shifting to more plant-based diets impacts the adequacy of protein intake in older adults needs to be studied.

**Objectives:**

We simulated how a transition towards a more plant-based diet (flexitarian, pescetarian, vegetarian, or vegan) affects protein availability in the diets of older adults.

**Setting:**

Community.

**Participants:**

Data from the Dutch National Food Consumption Survey 2019–2021 of community-dwelling older adults (n = 607) was used.

**Measurements:**

Food consumption data was collected via two 24-h dietary recalls per participant. Protein availability was expressed as total protein, digestible protein, and utilizable protein (based on digestibility corrected amino acid score) intake. The percentage below estimated average requirements (EAR) for utilizable protein was assessed using an adjusted EAR.

**Results:**

Compared to the original diet (∼62% animal-based), utilizable protein intake decreased by about 5% in the flexitarian, pescetarian and vegetarian scenarios. In the vegan scenario, both total protein intake and utilizable protein were lower, leading to nearly 35% less utilizable protein compared to the original diet. In the original diet, the protein intake of 7.5% of men and 11.1% of women did not meet the EAR. This slightly increased in the flexitarian, pescetarian, and vegetarian scenarios. In the vegan scenario, approximately 60% had a protein intake below EAR.

**Conclusions:**

Replacing animal-based protein sources with plant-based food products in older adults reduces both protein quantity and quality, albeit minimally in non-vegan plant-rich diets. In a vegan scenario, the risk of an inadequate protein intake is imminent.

## 1 Introduction

The current food system largely contributes to adverse environmental changes, mainly through the high usage of water and land, as well as the emission of greenhouse gases [1]. Meat and dairy, the main protein sources in an omnivorous diet, contribute the most to the food-related ecological footprint. A transition to a more sustainable diet-one that contains more plant-based products and less meat, could increase food availability while releasing the burden on the environment [2]. Furthermore, research suggests that a higher adherence to sustainable diets could lower the morbidity of chronic diseases, including cardiovascular diseases, type 2 diabetes, and certain cancers [3]. Currently, the mean ratio between animal-based/plant-based protein in the diets of Dutch inhabitants (1–79 years) is 57/43% [4], while it is advised to increase these values to 40/60% [5].

When transitioning to a more sustainable diet, special attention should be given to dietary protein needs. Plant-based proteins are generally lower in protein quantity and quality, the latter because of poor digestibility and amino acid composition [6,7]. Protein quality is an important factor in determining how much of the ingested protein is utilizable by the body. The quality of a protein source is determined by the digestibility and proportion of indispensable amino acids (IAA) combined, known as the Protein Digestibility–Corrected Amino Acid Score (PDCAAS) [8].

For older adults, consuming sufficient protein is of great importance as it prevents them from, among others, losing muscle mass [9]. When transitioning to a more sustainable diet, they might become at risk for an inadequate protein intake if either protein quantity or quality is insufficient. Because older adults often suffer from early satiation and have a lower appetite in general; the lower protein quantity, quality and higher fibre content in plant-based products pose challenges to reaching a sufficient intake of high-quality protein [10].

Currently, it remains uncertain whether older adults can meet their protein requirements through a more sustainable diet. A previous study among the general Dutch adult population (age 18–79) showed that sufficient protein intake is feasible when shifting from a 40% towards a 60% plant-based protein scenario [11]. Another simulation study performed on data from the Dutch National Food Consumption Survey (DNFCS) (2007–2010) in a younger adult population (18–65 years) showed a decrease in protein intake of about 20% in a “no meat, no dairy” dietary pattern. Nevertheless, nearly no one fell below the Estimated Average Requirements (EAR). Notably, protein quality and digestibility were not incorporated in these analyses and the analyses were restricted to adults aged under 65.

In this manuscript, we present the results of a simulation study on the diets of older adults (age ≥65 years) and evaluate the impact of the transition to a more sustainable eating pattern on protein quantity and quality. We explored various scenarios, including, flexitarian, pescetarian, vegetarian, and vegan dietary patterns to calculate quantity and quality of protein intake based on amino acid score and digestibility.

## 2 Methods

### 2.1 Study design

Data from the Dutch National Food Consumption Survey (DNFCS 2019–2021) was used [12]. The DNFCS is a periodical, cross-sectional food consumption survey, conducted by the National Institute for Public Health and The Environment (RIVM) [12]. Recruitment was done by a market research agency (Kantar) and the survey population was designed to be a representative sample of Dutch inhabitants. In 4-weekly waves, invitations were sent to nationally representative stratified random samples based on the following sociodemographic factors: age, gender, region, degree of urbanization and education level. In each wave, the composition and size of the sample were adjusted based on the response obtained in the previous waves. In total, 9.701 people were invited to participate of whom 3.570 (response rate 36.8%) individuals aged 1–79 were included in the DNFCS 2019 – 2021. In the population aged 65–79, 1701 people were invited, 607 of whom were included [12]. For this study, only that group was used. Besides food consumption, self-reported data on age, sex, height, weight, level of education and employment, and lifestyle characteristics were collected.

The Utrecht University Medical Ethical Review Committee evaluated the study and concluded it was not subject to the Medical Research Involving Human Subjects Act (WMO) of the Netherlands (reference number 19-145/C)

### 2.2 Dietary intake and amino acid composition

Food consumption data was collected via two non-consecutive 24-h dietary recalls per participant using the GloboDiet program [13]. Both recalls were performed within 2–6 weeks of each other. Participants aged 70 or older were asked to fill in a food diary on two specified days (days before the interview) which was only used as a memory aid for the 24-h recall. Participants aged between 65 and 70 were interviewed twice via telephone, while individuals aged 70 or older were interviewed once at home and once via telephone. Because of data collection during the COVID-19 pandemic, some were interviewed twice via telephone. Interviews were done in all seasons and on all days of the week. Food consumption was categorized by food consumption occasion (three meals and four in between moments) category by the participants.

Protein intake was calculated based on the amino acid dataset developed by Wageningen University and Research. This dataset is based on the Danish (Frida), American (USDA), English (McCance and Widdowson) and Japanese food composition tables and previously described by Heerschop et al. 2023 [14]. In this dataset, both amino acid content per food item and protein digestibility factors per food group (n = 27) are available.

As transitioning towards a more sustainable dietary pattern can influence both quantity (total protein content), as well as quality (digestibility and amino acid composition), we calculated protein intake/availability in three different ways: total protein intake (quantity), available protein intake after digestibility and utilizable protein intake based on the PDCAAS (quality).

Protein intake was derived by multiplying the consumed quantity by the total amino acid content of each food item and summing these overall food items. For the protein content after digestibility, we multiplied the total amino acid content of each food item by its specific digestibility factor.

We used amino acid content to calculate total protein intake. Not every amino acid contains the same amount of nitrogen and not all nitrogen in food items is bound in protein [15]. Thus, the standard conversion factor as applied in food composition databases (nitrogen from chemical analyses, multiplied by 6.25 (standard nitrogen content) or 6.38 (dairy products [16]) is too high for most food groups, leading to an overestimation of protein when applying the standard conversion factor [15].

Utilizable protein intake based on the PDCAAS was calculated per meal occasion, as optimal combinations of IAA can only be used if these IAAs are eaten within a limited time frame (i.e., one meal) [17,18]. Those IAAs that cannot be used will likely be directly oxidized [19]. We calculated the sum of amino acids after digestibility for each meal occasion (3 main meals and 4 in-between-meal moments). We then evaluated the similarity factor of the available IAAs in each meal to the WHO reference protein set by dividing the amino acid content per gram protein of the meal by the WHO reference protein values [20]. The similarity factor was determined by the most limiting amino acid. If there was no limiting amino acid during a meal, the similarity factor was maximized at 100%. The similarity factor was then multiplied by the total amount of amino acids after digestibility for that meal/snack moment. For daily utilizable protein intake, we calculated the sum of all separate meal/snack moments.

The Dutch food composition database (NEVO) [21] was used to assess the ratio of plant- vs. animal-based protein in the scenarios, as the amino acid database does not contain data on the source of protein.

### 2.3 Simulation of dietary scenarios

To assess the impact of a transition towards a more sustainable food pattern, different scenarios were created, representing two flexitarian (40%/80% of the meat replaced), one pescetarian (no meat, but fish and other animal-based products are included), one vegetarian (no meat and fish, but other animal-based products are included) and one vegan dietary scenario (no fish, meat, and animal-based products are included).

First, all products of the DNFCS were checked for the presence of values in the amino acid database; those without information (53 out of 1816 items) were replaced with comparable products by a dietitian (JWB) or nutritionist based on protein content and product similarity. For instance, boiled hare was replaced by boiled rabbit. Thereafter, all unique products used in the DNFCS were assessed to check if they met the criteria for the pescetarian, vegetarian or vegan dietary scenarios. Next, food products that did not fit into a scenario were classified by the same dietitian (JWB) and nutritionist into a food group and suitable alternatives were sought, based on the type of food (same food groups) and the context in which these products are eaten (see Table 1, Table 2). Replacement products were judged on adequacy and agreed upon by all co-authors (all of whom are either nutritionists or registered dietitians).

**Table 1:** Vegetarian alternatives used for replacements shown per food group, with energy and protein content per 100 grams.

Name	Protein/100 gram	kcal/100 gram	Name	Protein/100 gram	Kcal/100 gram
***Meat or fish***			***Sandwich fillings based on meat or fish***		
Chicken egg	12.3	128	Egg salad	7.3	255
Mozzarella cheese	18.7	253	Gouda cheese 48+	22.9	369
Vegetarian hamburger	17.7	192	Peanut butter	20.0	651
Vegetarian schnitzel	15.2	226	Spread sweet average	2.7	393
Plant based burger beyond meat	15.0	252	Hummus	7.7	322
Vegetarian meatballs	17.1	169	Vegan luncheon	8.2	156
Vegetarian burger with cheese	15.9	231	Cheese salad	9.0	422
Vegetarian meatball	16.9	243	Cream cheese	5.3	291
Valess schnitzel	12.3	188	Chicken egg	12.3	128
Gouda cheese 48+	22.9	369	Cheese 30+	31.9	306
Vegetarian sausage	13.3	259	Cheese spread	14.3	231
Vegetarian burger	11.0	210	Chocolate sprinkles	5.8	446
***Bread with meat***			***Soup***		
Cheese pastry	9.2	579	Soup clear w vegetables and noodles	0.7	17
Baguette cheese-onion	10	242	Soup clear vegetables	0.4	10
Bread currant	7.8	273	Soup thickened vegetables	0.7	36
Roll white soft	9.7	262	Soup thickened no filling	0.6	40
***savoury snack***			***Minced meat***		
Crisp potato	6.3	538	Lentils green/brown boiled	21.0	306
Cocktail snacks nibb-it	3.3	482	Quorn minced	13.0	97
Cassave crackers	1.4	474	Vegetarian minced meat	24.3	*124*
Falafel unprepared	6.8	208	Quorn pieces	14.0	114
			Mincemeat beyond meat	17.0	252
			Vegan mincemeat beans	17.0	182

Note: protein and kcal are based on Dutch food composition table (NEVO) [21].

**Table 2:** Vegan alternatives used for replacements shown per food group, with energy and protein content per 100 grams.

Name	Protein/100 gram	kcal/100 gram	Name	Protein/100 gram	Kcal/100 gram
***Meat, fish, Meat replacements based on dairy/egg, eggs (dinner), cheese (dinner)***			***Sandwich fillings based on meat, fish, cheese, eggs (breakfast and lunch)***		
lentils green/brown boiled	21	306	Peanut butter	20.0	651
Plant based burger beyond meat	17	252	Luncheon meat quorn	7.1	89
Falafel	6.8	208	Hummus natural	7.7	322
Vegan bean burger	16.0	191	Vegan bacon	5.5	140
Sausage beyond meat	17.0	233	Vegan luncheon meat	8.2	156
Tahoe soya curd	11.6	113	Vegan grill sausage	8.3	163
Vegan chicken burger	13.0	220	Sesame paste tahin	21.9	602
Plant based chicken vivera	15.0	166	Melt me smoky cheese	8.3	270
Vegan chicken	21.0	142	Vegan bacon strips	5.0	170
Vegan swedish balls	9.6	158	Vegan chicken filet	8.3	165
Vegan vegetable burger	11.0	116	Vegan sausage	7.0	244
Plant based chicken tenders	15.0	202	Spread sweet averaged	2.7	393
***Bread with dairy or meat***			***Drink with dairy***		
Roll white soft	9.7	262	Tea	0	0
Bread currant	7.8	273	Coffee	0	0
Bread brown/wholemeal	10.5	235	Drink soya original alpro	3	39
Bread rye dark	5.6	193	Water	0	0
***Yoghurt, cream sweet, custard, pudding, desserts, ice cream***			***Minced meat***		
Plant based alternative quark	2.7	119	Lentils green/brown boiled	21.0	306
Plant based alternative quark soya	5.7	54	Quorn minced	13.0	97
Yoghurt soybased fruit	3.7	134	Vegetarian minced meat	24.3	*124*
Havergurt greek style	3.3	145	Quorn pieces	14.0	114
Yoghurt soybased	4.0	47	Mincemeat beyond meat	17.0	252
Havergurt blue berry	1.3	101	Vegan mincemeat beans	17.0	182
Drink soya original alpro	3.0	39	***savoury snack***		
Original plant-based not milk	2.2	34	Crisp potato	6.3	538
Yoghurt soybased vanilla	3.7	134	Cocktail snacks nibb-it	3.3	482
Dessert soya alpro	3.1	85	Cassava crackers	1.4	474
Vly unsweated	2.5	34	Falafel unprepared	6.8	208
Ice cream coconutmilk	1.2	85	***Sweet snacks***		
***soup***			Turkish delight	0	*370*
Soup clear w vegetables and noodles	0.7	17	Popcorn sweet, popped wo oil	9.9	389
Soup clear vegetables	0.4	10	Chocolate dark w hazelnuts	9.0	565
Soup thickened vegetables	0.7	36	Wafer galette	6.9	480
Soup thickened no filling	0.6	40	***cream***		
			Cream based on veg oil alpro cuisine	2	166
			Cream based on veg oil Alpro Cuisine Light	2	63

Note: protein and kcal are based on Dutch food composition table (NEVO) [21].

In the vegan scenario, obvious animal-based products – cheese, milk, eggs – were marked as unfitting in the diet. Products that possibly include animal-based ingredients, such as cake and chocolate sprinkles were left unchanged, as they have minimal impact on daily protein intakes. For each of the products that needed to be replaced in one or more dietary scenarios, a dietitian selected up to 12 different alternatives from the same food group. If information about the IAA content of less than 12 suitable replacements was available, fewer replacements were used for that food group. Replacements with comparable products were done in gram-for-gram substitution.

Each food product to be replaced was randomly replaced by one of the available alternatives using a random number generator. The flexitarian diets (flexitarian-40 and flexitarian-80) were simulated in two steps: first, 40% or 80% of all meat and fish products were randomly selected for replacement. Thereafter, the random number generator was used to replace those items with alternatives.

Table 1 shows the vegetarian alternatives, which were used in the pescetarian, flexitarian and vegetarian diets for each food group. Table 2 shows the vegan alternatives.

### 2.4 Data analysis

All analyses (simulations and protein/amino acid calculations) were performed with R version 4.2.3. Characteristics of included participants of the DNFCS were described using descriptive statistics. Continuous variables are presented either as mean and standard deviation (SD) or as median and interquartile range (IQR) while categorical variables are given in frequencies and percentages.

The habitual intake (total protein intake, available protein after digestibility utilizable protein intake) for each scenario was calculated using the Statistical Program to Assess Dietary Exposure (SPADE) [22]. Weighting factors (sociodemographic factors, week/weekend days, season) were used to make data nationally and seasonally representative. A one-part model for daily intakes was used, and results are shown as median intake (p50) with IQR (p25–p75). Graphs were made by using Graphpad 10.2.3.

For traditional calculations of adequate protein intake, an Estimated Average Requirement (EAR) of 0.66 gram of high-quality protein per kilogram of body weight per day (g/kg bw/day) is used. This is based on nitrogen balance studies that do not consider digestibility [23]. Even high-quality protein sources like meat and dairy can result in some protein loss due to digestibility (digestibility factor 0.90−0.95) [7]. Since the EAR does not incorporate digestibility and amino acid composition, it is unsuitable for evaluating utilizable protein intake based on PDCAAS. In addition, the EAR is not applicable for total intake in situations where quality is lower [23], as in our more plant-based scenarios.

As there is no guideline to assess the EAR based on the PDCAAS, we recalculated the standard EAR of 0.66 g/kg bw/day. We assessed which percentage of protein intake is lost due to protein quality. To do this, we used the original diet assuming that the quality in our data is comparable to the original nitrogen balance studies [23]. The median loss between total protein intake and utilizable protein intake based on PDCAAS in the original diet, 11.9% (IQR: 9.6–14.9%), was subtracted from 0.66 g/kg bw/day, resulting in an adjusted EAR of 0.58 g/kg bw/day as the reference value to estimate the prevalence of the population with adequate PDCAAS intake.

## 3 Results

In total, 607 participants were included, half of them being men. The median age was slightly higher in men (71 years) compared to women (69 years), and BMI was around 27 kg/m^2^ in both groups (Table 3). Men were more frequently highly educated (41% high) than women (27% high).

**Table 3:** Characteristics of the participants and five more sustainable scenarios from the Dutch National Food Consumption Survey 2019-2021 aged 65 or higher stratified by sex.

Characteristic	Men *n* = 311 (51%)	Women *n* = 296 (49%)
Age (Years)	71 (68–84)	69 (67–73)
BMI (kg/m2)	26.9 (±4.5)	26.8 (±5.4)
Education level (n; %)		
Low	96 (31%)	132 (45%)
Middle	89 (29%)	83 (28%)
High	126 (41%)	81 (27%)
Number of food replacements		
Flexitarian-40	771	596
Flexitarian-80	1405	1010
Pescetarian	1355	901
Vegetarian	1709	1227
Vegan	4754	4058
Percentage plant-based protein of total protein		
Original diet	39.0%	37.7%
Flexitarian-40	43.7%	42.6%
Flexitarian-80	52.2%	49.7%
Pescetarian	54.3%	51.9%
Vegetarian	59.1%	54.2%
Vegan	99.3%	99.3%
Kcal intake		
Original diet	2188 (1947–2455)	1741 (1539–1956)
Flexitarian-40	2220 (1982–2481)	1761 (1560–1973)
Flexitarian-80	2252 (1998–2532)	1791 (1583–2011)
Pescetarian	2258 (2009–2538)	1786 (1581–2002)
Vegetarian	2278 (2023–2561)	1799 (1591–2018)
Vegan	2161 (1918–2429)	1726 (1530–1932)

Data is shown as number (%), median (IQR) or mean (SD). Note that the vegan scenario did not lead to 100% plant-based protein as products with a low amount of animal protein, such as cakes, were not replaced.

The lowest frequency of a food replacement was needed in the flexitarian-40 scenario (1367 times), while the highest frequency was needed to create the vegan scenario (8812 replacements). In the reference scenario, the plant-based proportion of total protein intake was 39.0% in men and 37.7% in women. In the vegetarian scenario, the plant proportion of total protein intake was 59.1% in men and 54.2% in women, while in the vegan scenario, plant-based protein was nearly 100%. Total energy intake remained relatively stable (Δ<100 kcal) for all scenarios in both genders.

**Fig. 1:**
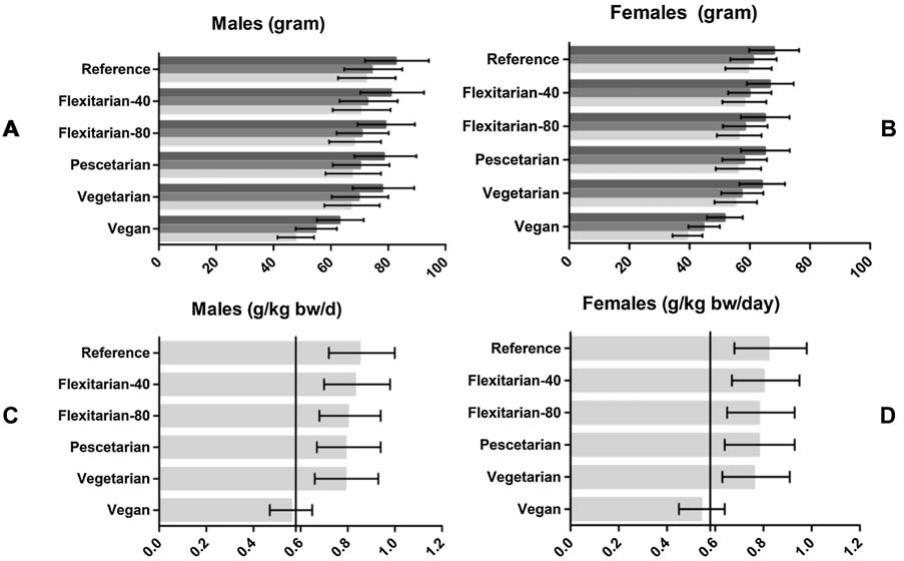
Daily median protein intake, protein intake after correction for digestibility and utilizable protein intake of older adults (≥65 year) in the Dutch National Food Consumption Survey, stratified by gender and scenario. *Data on X-axes are shown as median daily protein intake in grams and g/kg bw/day with error-bars representing 25th and 75th percentile. A: Daily intake of men (gram), B: daily intake of Women (gram), C: Daily intake of men (g/kg bw/day), D: Daily intake of women (g/kg bw/day)*

The median habitual daily protein intake in the original diet was 82.5 grams (0.96 grams/kg bw/day) for men and 67.9 grams (0.94 grams/kg bw/day) for women (see Fig. 1A/B). Compared to the original diet, the difference in daily protein intake was relatively low in most scenarios (2–6%) except in the vegan scenario where loss amounted up to 25% (62.9 grams for men and 51.5 grams for women).

Loss of protein due to digestibility was comparable for most scenarios (about 10%), but slightly higher in the vegan scenario (14%). In the flexitarian-40/80 pescetarian and vegetarian scenarios, utilizable protein intake was comparable with intake based on digestible protein. However, in the vegan scenario, utilizable protein intake was approximately 13% lower than intake based on digestible protein.

Compared to the original diet, utilizable protein intake in the vegetarian scenario differed by about 5 grams in men and 3 grams in women, whereas in the vegan scenario, this difference was about 25 grams in male and 20 grams in female.

In the original diet, the median utilizable protein intake per kg bodyweight per day was 0.85 in men and 0.82 in women (Fig. 1C/D). This was lower for each scenario with higher ratios of plant-based protein and was 0.79 (men) respectively 0.77 (women) in the vegetarian scenario. However, a larger difference was seen in the vegan scenario where the utilizable protein intake was 0.56 g/kg bw/day in males and 0.54 g/kg bw/day in females.

In the reference scenario, the prevalence of intake below EAR (utilizable protein intake below 0.58 g/kg bw/day) was 7.5% in men and 11.1% in women. This was to 12.5% and 16.4% in the vegetarian scenario while in the vegan scenario, over half of older adults had an intake below EAR (56.7% in males and 60.2% in females).

**Fig. 2:**
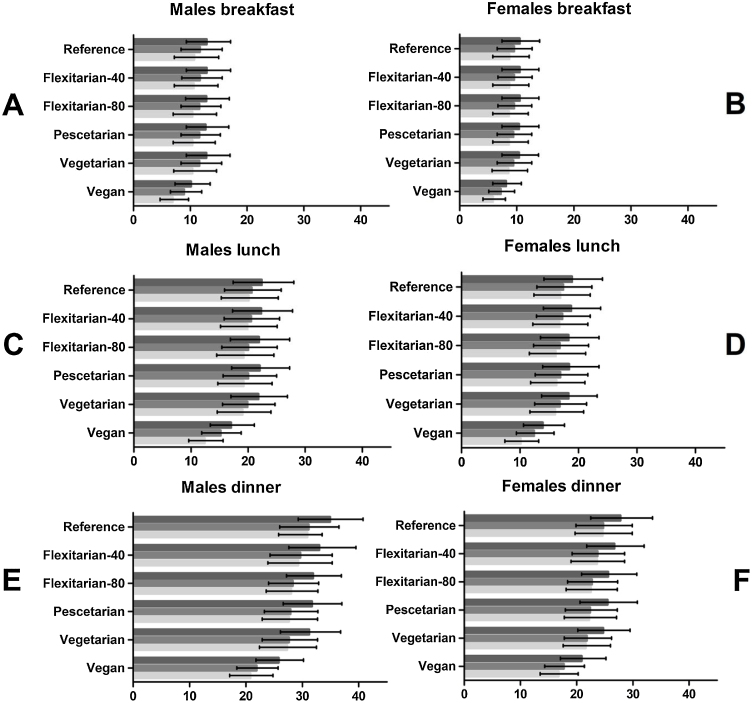
Median protein intake, protein intake after correction for digestibility and utilizable protein intake of older adults (≥65 year) in the Dutch National Food Consumption Survey, stratified by gender, eating occasion and scenario. *Data on X-axes are shown as median protein intake in grams, with error bars representing 25th and 75th percentile. A: breakfast intake of men, B: breakfast intake of Women, C: lunch intake of men, D: lunch intake of men, E: Dinner intake of men, F: Dinner intake of women. Only mean meals are shown as they provide the largest part of protein.*

Fig. 2 depicts protein intake and availability per meal moment. The median quantity of protein intake at breakfast was, depending on the scenario, approximately 10−13 grams of protein for men and 8−11 grams for women (Fig. 2A/B). Differences between scenarios were small. At breakfast, the proportion of protein that cannot be utilized due to digestibility or amino acid composition was 20% in most scenarios, but over 30% in the vegan scenario.

For lunch, intake in the original diet, flexitarian-40, flexitarian-80, pescetarian and vegetarian scenario was relatively comparable with approximately 22 grams of protein for men and 18 for women (Fig. 2 C/D). In the vegan scenario, this was 17 and 14 grams. At lunch, the proportion of protein that cannot be utilized due to digestibility or amino acid composition was in the original diet, flexitarian-40, flexitarian-80, pescetarian and vegetarian scenario smaller than at breakfast (10−13%) but in the vegan scenario, this loss due to quality was again around 30%.

The highest median protein intake was observed at dinner (Fig. 2E/F). In agreement with breakfast and lunch, differences in intake between most scenarios (except vegan) were small and men consumed 31−35 grams and women, 25−28 grams. Again, the lowest intake was seen in the vegan scenario with 26 grams for men and 21 grams for women. At dinner, the proportion of protein that cannot be utilized due to digestibility or amino acid composition was 10−13% in most scenarios and 20% in the vegan scenario

## 4 Discussion

This study aimed to assess how protein quantity and quality differ between the present dietary intake and various more plant-based eating patterns in Dutch older adults aged between 65 and 79. In our simulated diets, protein quality and quantity decreased with increasing proportions of plant-based protein. However, in all non-vegan scenarios, protein quantity and quality did not show large reductions. The vegan diet however led to severely lower protein quantity, especially when correcting for protein quality, leading to 60% of older consumers not meeting the protein requirements.

The strong decrease in the vegan scenario was expected because of the low quantity and quality of plant-based products [6,7]. Only one previous study (French general population, 18–65 years n = 1678) simulated a full vegan pattern while incorporating protein quality [24]. This study adopted a more theoretical approach by substituting animal-based protein with plant-based protein without replacing specific products. It reported a decrease in utilizable protein intake, but the percentage of the population falling below the EAR was small, suggesting that a shift towards a vegan pattern is safe [24]. However, in their original diet, protein intake was much higher (∼1.30 g/kg bw/day) compared to what was observed in our study (∼0.85 g/kg bw/day).

A previous “no meat and no dairy diet” simulation study (eggs and fish were still allowed) performed on the DNFCS 2007–2010 led to a reduction of 20% of protein intake [25], which was 25% in our vegan scenario. Differences may be related to protein quality, which was taken into account in our study, but not in the previous one.

Previous cross-sectional studies of vegetarian and vegan eating patterns -mostly conducted in the general adult population (18–65 years)- showed little to no risks from switching towards a vegetarian or vegan dietary pattern [26]. Protein intake was usually lower in the vegan group, but as the general intake in these studies was high, the vegan protein intake remained above the required levels. This could be explained by the fact that people who follow a vegan diet today are relatively aware of their health and food intake [27]. In addition, most previous studies did not consider digestibility and amino acid composition, whereas we have shown that -in a predominantly plant-based diet- the additional loss due to lower quality is nearly as large as the loss due to lower quantity. Therefore, we expect that studies of high plant-based protein diets that only assess total intake without considering digestibility and amino acid composition are likely to overestimate protein availability.

To our knowledge, only one cross-sectional study from Denmark has assessed protein quality using amino acid composition. This study (n = 40, age 16–59 years) showed that more than half of the measured daily requirements for either total protein or amino acids were not met. In particular, those with a low energy intake were at risk of low intakes [28].

Our vegetarian scenario nearly reached the recommended level of 60% plant-based protein [5] without a significant decline in loss of utilizable protein intake. This implies that older adults can maintain an adequate protein intake while eating less meat/fish. This is in line with previously performed simulation studies [29]. Notably, protein intake in our vegetarian scenario was comparable to the flexitarian/pescetarian scenarios. In all these scenarios, loss due to digestibility and amino acid composition was low. This is in line with a previous study that showed that protein quality is only a factor of interest when plant-based protein is above 70% [24].

The loss in protein intake (quantity) in all scenarios was mainly observed at dinner. This was expected as most protein during that meal moment comes from meat [30], which we replaced with alternatives that sometimes had lower protein quantities. The loss in protein quality was greatest at breakfast and lunch. During these meal occasions, 50–60% of protein comes from plant-based products [30], mostly bread, short in lysine. In the original diet, animal-based products compensate for the lower quality. This compensation mechanism ceases when we replace animal products with plant-based ones that have much lower protein quality. To compensate for the low lysine content of one slice of bread (35 grams) one would need to combine it with, for example, 155 grams of hummus (beans, relatively rich in lysine) or one glass (200 ml) of soy milk.

Traditional calculations of protein, based on the Dutch food composition table [31], showed a median protein intake of 84.1 g/day for men and 73.1 g/day for women. In our calculations, based on an amino acid database, this was slightly lower, with 82.5 g/day and 67.9 respectively. As described in the methods section, this could be because of different methodologies of assessing the protein/amino acid content of products. However, it remains unclear why this difference was larger in women than in men.

Our study represents the first comprehensive assessment of protein availability specifically focused on older adults. As we used a nationally representative and relatively large dataset, our study provides a good overview of how the protein intake of older adults may be affected by a transition towards a more sustainable diet. The main strength of our study is the assessment of protein availability by incorporating both digestibility and amino acid composition. While most previous studies have focused solely on quantity [29], we highlight that, particularly in the vegan scenario, the adjustments for digestibility and quality are of equal importance. Another strength is the replacement strategy that we used. We opted for a realistic scenario in which (up to 12) realistic replacements within different food categories were selected by a dietitian and nutritionist.

However, the choice of replacements is also a limitation because a simulation study may not always adequately reflect real-life practice: original food products are often not replaced with alternatives of the same weight as products differ in volume and have a different impact on satiation and satiety. Especially in the vegan scenario, high-fibre products are expected to lead to earlier satiation and thus to the intake of smaller volumes of products eaten. Furthermore, we chose scenarios with meat replacements that are processed and convenient in preparation. Higher protein quality per meal in the vegan scenario is possible when smart combinations are made by also replacing other meal components, real-life practice shows that people usually replace an animal-based product with a similar plant-based alternative, without making other changes within the same meal [32]. Finally, we used a very strict timeframe to assess protein quality (within one meal moment). Although it is assumed that protein should be eaten within a limited time frame [17,18] it is unclear exactly how long this period should be. Our timeframe might be too tight, leading to an underestimation of protein availability, especially in the vegan scenario. However, as mentioned before, lysine shortage especially at breakfast and lunch is so large that even daily, this is not likely to be fully compensated by other products [24].

Another consideration that needs to be made is the consequence of lower protein intake in older adults, for example on bone and muscle health, [33]. Will this impair protein synthesis and lead to muscle loss [34]? Our study only focussed on protein intake without assessing other outcomes on, for instance, micronutrient intake and sustainability. Whether micronutrient requirements can be met in more plant-based eating patterns needs further study [5]. We expect that the scenarios we simulated are more sustainable as they contain less animal-based protein, but we performed no lifecycle assessments as this was not within the scope of our research. Future studies should focus on these aspects. Finally, this field of research is progressing rapidly. The reliability and validity of working with PDCAAS need to be further established [35].

To conclude: we showed in this simulation study that replacing animal-based with plant-based protein sources in the diet of older adults led to reductions in protein quantity and quality only when all animal-based foods were eliminated. Shifting to a flexitarian, pescetarian or vegetarian diet only marginally influenced protein intake and did not place older adults at risk for an inadequate protein intake. Changing protein intake to 60% plant-based protein seems to be safe for older adults in terms of protein intake. In contrast, a vegan pattern was associated with a substantial decline in protein availability, leading to more than half of older adults not reaching the recommended protein levels

## Funding

This research was funded by the Taskforce for Applied Research, part of the Netherlands Organization for Scientific Research (NOW), financed by the Dutch Ministry of Education, Culture and Science AND by a fund of the Dutch Dairy Association. Funders had no role in the design, analyses and writing or the decision to submit the manuscript.

## Data availability

Data described in the manuscript, code book, and analytic code will be made available upon request pending [e.g., application and approval, payment, other].

## Declaration of competing interest

The authors declare that they have no known competing financialinterests or personal relationships that could have appeared to influencethe work reported in this paper.
